# Mitochondrial genome and phylogenomic analysis of *Pseudo-nitzschia micropora* (Bacillariophyceae, Bacillariophyta)

**DOI:** 10.1080/23802359.2021.1923426

**Published:** 2021-06-21

**Authors:** Yang Chen, Zongmei Cui, Feng Liu, Nansheng Chen

**Affiliations:** a CAS Key Laboratory of Marine Ecology and Environmental Sciences, Institute of Oceanology, Chinese Academy of Sciences, Qingdao, China; b Laboratory of Marine Ecology and Environmental Science, Qingdao National Laboratory for Marine Science and Technology, Qingdao, China; c University of Chinese Academy of Sciences, Beijing, China; d Center for Ocean Mega-Science, Chinese Academy of Sciences, Qingdao, China; e Department of Molecular Biology and Biochemistry, Simon Fraser University, Burnaby, British Columbia, Canada

**Keywords:** Mitochondrial genome, phylogenetic analysis, *Pseudo-nitzschia micropora*

## Abstract

The number of species in the genus *Pseudo-nitzschia* has increased to 56, including 26 species known to produce domoic acid (DA), which is harmful to marine animals and human health. The lack of genomic sequences of *Pseudo-nitzschia* species has been a limiting factor in the studies of genetic and evolutionary relationships of *Pseudo-nitzschia* species. Here, the complete mitochondrial genome sequence of *Pseudo-nitzschia micropora* was determined for the first time, which was 38,792 bp in length with the overall AT content being 69.98%. The mitochondrial genome encoded 62 genes, including 36 protein-coding genes (PCGs, including *orf157*), 24 transfer RNA (tRNA) genes and two ribosomal RNA (rRNA) genes. Phylogenetic tree analysis suggests that the *P. micropora* had a closer relationship with *P. cuspidate* than that with *P. multiseries*. The availability of the complete mitochondrial genome of *P. micropora* would be useful for researching the evolutionary relationships of *Pseudo-nitzschia* species.

Many species of the genus *Pseudo-nitzschia* are cosmopolitan and can form blooms in coastal regions, some of which can produce domoic acid (DA) that cause amnesic shellfsh poisoning (ASP), which is harmful to seabirds, marine mammals and human health (Scholin et al. 2000; Dong et al. 2020). As a genus in the order Bacillariales (Bacillariophyceae, Bacillariophyta), the number of described *Pseudo-nitzschia* species has increased to 56 (Dong et al. 2020), including 26 known to produce DA (Dong et al. 2020). Nevertheless, the genomic sequences of these species are limited. Such genomic sequences are critical for analyzing genetic and evolutionary relationships of species in the genus *Pseudo-nitzschia*. *Pseudo-nitzschia micropora* Priisholm et al. 2002 is a nontoxic species but has been reported to be high abundance during an algal bloom in Cuyutlan Lagoon (Colima, Mexico) (Quijano-Scheggia et al. 2011). It has been identified in many countries including Thailand (Priisholm et al. 2002), Vietnam (Larsen and Nguyen 2004), Malaysia (Lim et al. 2012), Korea (Park et al. 2009) and China (Xu and Li 2015). In this study, we constructed the complete mitochondrial genome of *P. micropora.* The strain CNS00133 analyzed in this study was isolated from water sample using single cell capillary method, during an expedition to the Jiaozhou Bay (36°09.472′N, 120°15.055′ E) in August 2019 on the research vehicle ‘Innovation’, and its specimen was deposited in the collection of marine algae in KLMEES of IOCAS (Nansheng Chen, chenn@qdio.ac.cn) with the voucher number CNS00133. Briefly, after 2 weeks in culture, the algal cells were harvested by centrifugation (12,000 g, 5 min) and stored in liquid nitrogen for the next DNA extraction step.

Total genomic DNA of *P. micropora* was extracted using a TIANGEN DNAsecure Plant Kit (Tiangen Biotech, China) according to the manufacturer’s instructions. The genomic DNA library was sequenced using Illumina NovaSeq 6000 platform (Illumina, USA). Illumina sequencing results (about 5 Gb) of *P. micropora* were assembled into the mitochondrial genome using GetOrganelle v 1.7.2 (Jin et al. 2020), with SPAdes v 3.13.2 as the assembler (Bankevich et al. 2012). The completed mitogenome of *P. micropora* was circular in shape and 38,792 bp in length (Genbank accession number MW423602), which was slightly longer than that of *P. cuspidate* (37,203 bp), but much shorter than that of *P. multiseries* (46,283 bp) (Yuan et al. 2016). The overall AT content of the mitogenome of *P. micropora* was 69.98%, which was similar to that of *P. cuspidate* (69.83%) and *P. multiseries* (68.95%) (Yuan et al. 2016). The *P. micropora* mitogenome encoded 62 genes, including 36 protein-coding genes (PCGs, including *orf157*), 24 transfer RNA (tRNA) genes and two ribosomal RNA (rRNA) genes. The length of *cox1* (1,512 bp) of *P. micropora* was identical to that of *P. cuspidate*. No introns were found in any genes of the *P. micropora* mitogenome. However, the *tatA* gene of *P. micropora* (129 bp) was much shorter than that of *P. cuspidata* (246 bp) and *P. multiseries* (234 bp). These three mitogenomes of the genus *Pseudo-nitzschia* shared 34 PCGs, while *rps7* is absent from the mitochondrial genome of *P. multiseries*. There was no difference among the number and category of the tRNAs and rRNAs between three mitochondrial genomes, respectively.

Maximum likelihood (ML) phylogenetic tree was constructed using tandem amino acid sequences of 31 common protein-coding genes (*atp6*, *8*, *9*; *cob*; *cox1*, *2*, *3*; *nad1*-*7*, *4 L*, *9*, *11*; *rpl2*, *5*, *6*, *14*, *16*; *rps3*, *4*, *8*, *10*, *11*, *13*, *14*, *19*; and *tat*C) shared by 40 species (Figure 1) using IQtree v1.6.12 (Trifinopoulos et al. 2016) with 1000 bootstrap alignments. Two Oomycota species *Phytophthora Ramorum* (EU427470) and *Saprolegnia Ferax* (NC_005984) were used as out-group taxa. The 38 Bacillariophyta species clustered well into three clades corresponding to three classes Coscinodiscophyceae, Mediophyceae and Bacillariophyceae. *P. micropora* clustered well with *P. cuspidate* and *P. multiseries* with strong support (Figure 1). *P. micropora* had a closer relationship with *P. cuspidate* than *P. multiseries*.

**Figure 1.**
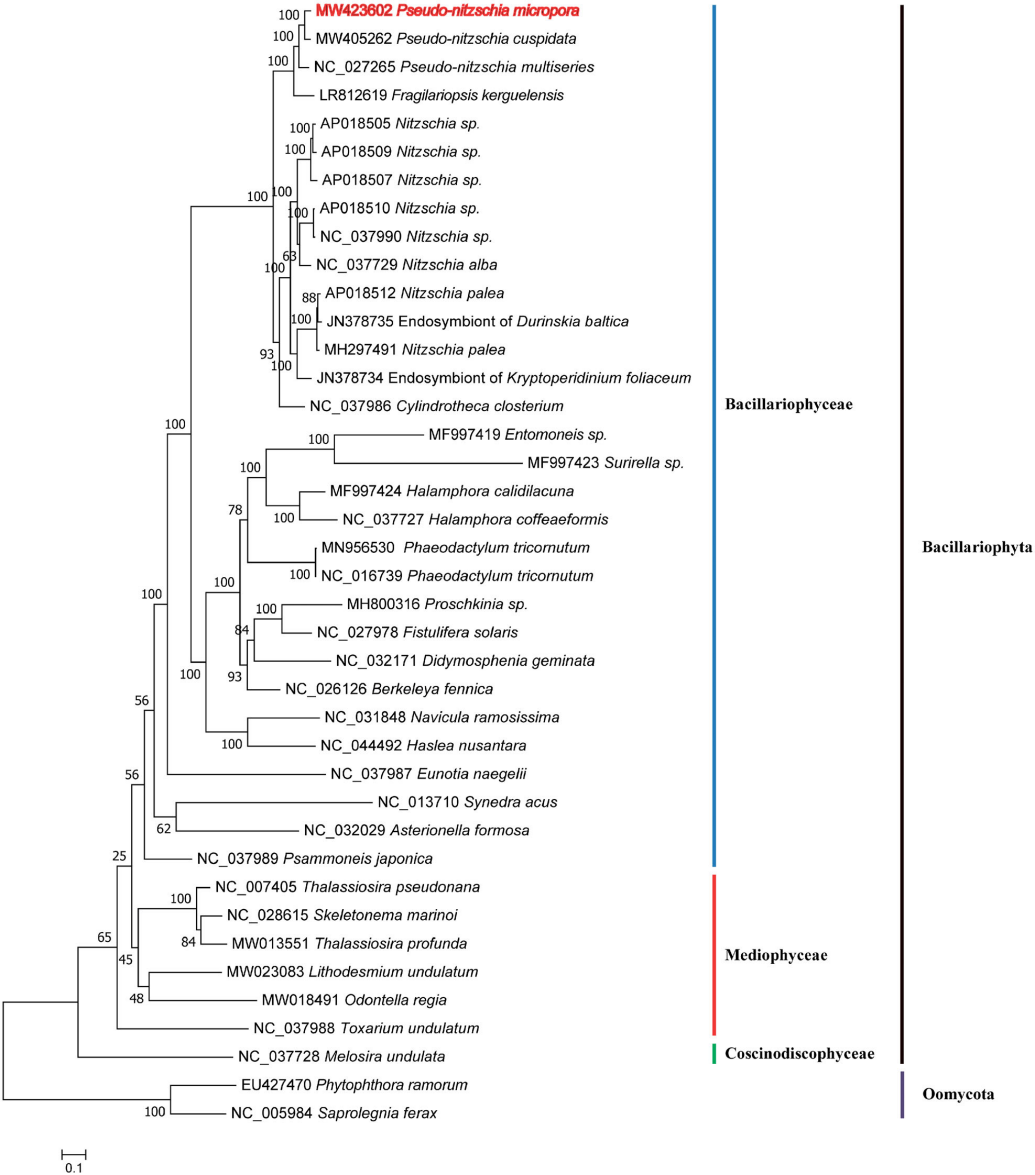
Maximum likelihood (ML) tree based on 31 PCGs of 38 Bacillariophyta species plus two Oomycota species as outgroups. GenBank accession numbers are given before the species name.

The construction of the mitochondrial genome of *P. micropora* was not only valuable for studies on genetic diversity of this species, but also could contribute to further understanding of the evolutionary relationships of *Pseudo-nitzschia* species.

## Data Availability

The genome sequence data that support the findings of this study are openly available in GenBank of NCBI at https://www.ncbi.nlm.nih.gov/nuccore/MW423602, under the accession no. MW423602. The associated BioProject, SRA and Bio-Sample numbers are PRJNA687399 (https://www.ncbi.nlm.nih.gov/bioproject/PRJNA687399), SRR13319799 (https://www.ncbi.nlm.nih.gov/sra/SRR13319799) and SAMN17173859 (https://www.ncbi.nlm.nih.gov/biosample/ SAMN17173859), respectively.
